# Assessment of sesquiterpene lactones isolated from *Mikania* plants species for their potential efficacy against *Trypanosoma cruzi* and *Leishmania* sp.

**DOI:** 10.1371/journal.pntd.0005929

**Published:** 2017-09-25

**Authors:** Laura C. Laurella, Natacha Cerny, Augusto E. Bivona, Andrés Sánchez Alberti, Gustavo Giberti, Emilio L. Malchiodi, Virginia S. Martino, Cesar A. Catalan, María Rosario Alonso, Silvia I. Cazorla, Valeria P. Sülsen

**Affiliations:** 1 Universidad de Buenos Aires, Facultad de Farmacia y Bioquímica, Cátedra de Farmacognosia, Buenos Aires, Argentina; 2 CONICET—Universidad Nacional de Luján, Instituto de Ecología y Desarrollo Sustentable (INEDES), Luján, Argentina; 3 Universidad de Buenos Aires, Facultad de Farmacia y Bioquímica, Cátedra de Inmunología, Buenos Aires, Argentina, Instituto de Estudios de la Inmunidad Humoral (IDEHU), UBA-CONICET, Buenos Aires, Argentina; 4 CONICET- Universidad de Buenos Aires, Instituto de Microbiología y Parasitología Médica—CONICET (IMPaM), Facultad de Medicina, Piso 13, Buenos Aires, Argentina; 5 CONICET–Universidad de Buenos Aires, Instituto de Química y Metabolismo del Fármaco—CONICET (IQUIMEFA), Buenos Aires, Argentina; 6 CONICET–Universidad Nacional de Tucumán, Instituto de Química del Noroeste—CONICET (INQUINOA), Ayacucho 471 (T4000INI), San Miguel de Tucumán, Argentina; 7 CONICET–Centro de Referencia para Lactobacilos (CERELA), Batalla de Chacabuco 145, San Miguel de Tucumán, Argentina; Academic Medical Centre, NETHERLANDS

## Abstract

Four sesquiterpene lactones, mikanolide, deoxymikanolide, dihydromikanolide and scandenolide, were isolated by a bioassay-guided fractionation of *Mikania variifolia* and *Mikania micrantha* dichloromethane extracts. Mikanolide and deoxymikanolide were the major compounds in both extracts (2.2% and 0.4% for *Mikania variifolia* and 21.0% and 6.4% for *Mikania micrantha* respectively, calculated on extract dry weight). Mikanolide, deoxymikanolide and dihydromikanolide were active against *Trypanosoma cruzi* epimastigotes (50% inhibitory concentrations of 0.7, 0.08 and 2.5 μg/mL, for each compound respectively). These sesquiterpene lactones were also active against the bloodstream trypomastigotes (50% inhibitory concentrations for each compound were 2.1, 1.5 and 0.3 μg/mL, respectively) and against amastigotes (50% inhibitory concentrations for each compound were 4.5, 6.3 and 8.5 μg/mL, respectively). By contrast, scandenolide was not active on *Trypanosoma cruzi*. Besides, mikanolide and deoxymikanolide were also active on *Leishmania braziliensis* promastigotes (50% inhibitory concentrations of 5.1 and 11.5 μg/mL, respectively). The four sesquiterpene lactones were tested for their cytotoxicity on THP 1 cells. Deoxymikanolide presented the highest selectivity index for trypomastigotes (SI = 54) and amastigotes (SI = 12.5). In an *in vivo* model of *Trypanosoma cruzi* infection, deoxymikanolide was able to decrease the parasitemia and the weight loss associated to the acute phase of the parasite infection. More importantly, while 100% of control mice died by day 22 after receiving a lethal *T*. *cruzi* infection, 70% of deoxymikanolide-treated mice survived. We also observed that this compound increased TNF-α and IL-12 production by macrophages, which could contribute to control *T*. *cruzi* infection.

## Author summary

Chagas' disease and Leishmaniasis are parasitic diseases that affect especially poor people in developing countries. They are caused by the protozoan parasites *Trypanosoma cruzi* and different *Leishmania* species, respectively. According to the World Health Organization they are considered, among others, Neglected Tropical Diseases. The drugs currently in use for the treatment of these parasitoses are not at all effective and have severe drawbacks. Nature has proved to be a rich source of bioactive compounds. Among antiparasitic drugs, the sesquiterpene lactone artemisinin and the alkaloid quinine and their derivatives are used nowadays for human malaria treatment. Sequiterpenes lactones, present mainly in species from Asteraceae family, are interesting compounds due their pharmacological properties. This group of compounds has shown trypanocidal and leishmanicidal activity and are considered as promising leads for antiparasitic drug discovery.

## Introduction

Chagas’ disease is caused by the protozoan parasite *Trypanosoma cruzi*, and even though over 100 years have passed since its discovery, this disease still represents a serious problem for global health [1]. Thus, this disease, which was traditionally limited to Latin America, has crossed these boundaries and spread worldwide. Increased travelling and migration of individuals from endemic to non-endemic areas has presented a worrisome scenario for congenital infection, blood transfusions and organ transplantations [2, 3]. *Trypanosoma cruzi* infects a wide range of mammalian hosts, including humans. About six to seven million people are known to be affected by this infection [4]. The alarming numbers would be even greater if the underdiagnosed cases were taken in account. Vector control programs have not been completely successful in preventing parasite transmission [5].

Leishmaniasis is another protozoan parasitic disease caused by *Leishmania* spp., which presents a worldwide distribution. According to World Health Organization, 300000 cases of visceral leishmaniasis are reported annually, with 200000 deaths One million cases of cutaneous leishmaniasis have been reported in the last five years and 310 million people are at risk of acquiring this parasitosis [6]. Cutaneous leishmaniasis is the most common clinical form in Argentina caused by *L*. *(V*.*) braziliensis* and *L*. *(L*.*) amazonensis*, with some recent reports about human and canine leishmaniasis [7]. Available anti-parasitic drugs for both Chagas’ disease and leishmaniasis are not sufficiently safe or effective [8,9] and no protective vaccines have been developed so far [10].

Natural products are an interesting source of new drugs that might, in the near future, replace current medications, which are known to have severe side effects. Sesquiterpene lactones are a group of natural compounds, mainly found in the Asteraceae family, which show interesting biological activities such as antitumoral, antiinflammatory, antibacterial and antiparasitic, among others [11]. Some of the most representative compounds with antiparasitic activity are artemisinin, parthenolide, costunolide, helenalin, mexicanin, psilostachyins and cynaropicrin [12–14].

The *Mikania* genus (Asteraceae) is found in the tropics of America and Asia and many of its species which are known with the common name of “guaco” are used for treating fever, rheumatism, colds and respiratory diseases, as well as for snake bites and scorpion stings. In recent years, there has been an increasing interest in the study of species from the genus *Mikania*, since many of its species have been reported to have a wide range of bioactivities [15].

*Mikania variifolia* Hieron. and *M*. *micrantha* Kunth are species native to South America. These species grow in the Northeastern region of Argentina, South of Brazil, Paraguay and Uruguay. In particular, *M*. *micrantha* is considered a very invasive weed, growing in cattle fields [16] and has spread throughout Asia. It is used in popular medicine as vulnerary and as antidote [17]. In previous investigations, the isolation of sesquiterpene lactones, flavonoids and caffeoylquinic acids esters have been reported [18–21]. Antimicrobial and antiviral activities have also been described for this species [22]. We have previously reported the antiprotozoal and antiviral activities of extracts from *M*. *micrantha*. The organic extract of this species has proved to be active against *T*. *cruzi* epimastigotes and *Leishmania braziliensis* promastigotes. The bioassay-guided fractionation of this extract led to the identification of two fractions with trypanocidal activity which showed the presence of sesquiterpene lactones [23].

In this work we describe the isolation of the active constituents of *M*. *variifolia* and *M*. *micrantha* and the anti-*Trypanosoma cruzi* and antileishminial activity of the isolated compounds.

## Materials and methods

### Plant material

The aerial parts of *Mikania variifolia* Hieron. (Asteraceae) were collected in December 2012 in the province of Entre Rios, Argentina. A voucher specimen (BAF 788) was deposited at the Herbarium of the Museo de Farmacobotánica—Facultad de Farmacia y Bioquímica, Universidad de Buenos Aires. The aerial parts of *Mikania micrantha* Kunth (Asteraceae) were collected in April 2011 in the province of Tucumán, Argentina. A voucher specimen (LIL609699) was deposited at the Herbarium of Instituto Miguel Lillo, Facultad de Ciencias Naturales, Universidad Nacional de Tucumán.

### Parasites

*Trypanosoma cruzi* epimastigotes (RA strain) were grown in a biphasic medium. Cultures were routinely maintained by weekly passages at 28°C. *T*. *cruzi* bloodstream trypomastigotes (RA strain) and the recombinant Tulahuen strain expressing β-galactosidase (Tul-β-Gal) were obtained from infected CF1 mice by cardiac puncture at the peak of parasitemia on day 15 post-infection [24]. Trypomastigotes were routinely maintained by infecting 21-day-old CF1 mice.

*Leishmania braziliensis* promastigotes (MHOM/BR/75/M2903 strain) were grown in liver infusion tryptose medium LIT, which was prepared as follows: 5 g/L liver infusion (Sigma 2023-072K1066), 5 g/L tryptose (Britania), 2 g/L glucose (Sigma), 68 mM NaCl, 5.4 mM KCl, 22 mM HPO_4_Na_2_, supplemented with 20 mg/L hemin (Sigma) and 10% (vol/vol) fetal calf serum (FCS) (Internegocios). Culture maintenance was performed by weekly passages at 26°C.

### Preparation of extracts

The extraction of the aerial parts of *M*. *variifolia* (400 g) and *M*. *micrantha* (100 g) was done by maceration with dichloromethane (8L and 1L, respectively) at room temperature. The organic extracts were filtered and taken to dryness.

### Bioassay-guided fractionation of *M*. *variifolia* organic extract

The extract residue of *M*. *variifolia* was suspended in ethanol:water (70:30) (50 mL) and partitioned successively with hexane (3x40 mL) and dichloromethane (3x40 mL). The dichloromethane fraction (3.5 g) was subjected to open-column chromatography over silica gel 60 with a gradient of dichloromethane/ethyl acetate/methanol, collecting fractions of 70 mL each. Fractions were then combined according to their TLC profile on Silica gel 60 F254 using hexane:ethyl acetate (1:1) as mobile phase, into six final fractions (MV1–MV6). These fractions were subsequently tested for trypanocidal activity against *T*. *cruzi* epimastigotes. Compound 1 precipitated from fraction MV3 (eluted with dichloromethane:ethyl acetate (3:1). Compound 2 precipitated from MV4 eluted with dichloromethane:ethyl acetate (2:1). The supernatant from this fraction was subjected to chromatography on a Silica gel column eluted with hexane:ethyl acetate (1:1) and compound 3 precipitated from one of the fractions.

### Isolation of compounds from *Mikania micrantha*

The fractionation procedure of *M*. *micrantha* active extract was described previously [23]. The active fractions MM3 and MM4 were combined (1.25 g) and subjected to Silica gel column (51 cm x 3.5 cm, 110 g) chromatography eluted with a gradient of hexane, dichloromethane, ethyl acetate and methanol. Fractions of 20 mL were collected. Compound 1 and compound 2 precipitated from fractions eluted with dichloromethane:ethyl acetate (95:5) and dichloromethane:ethyl acetate (90:10), respectively. Compound 3 crystallized from the supernatant of fractions eluted with dichloromethane:ethyl acetate (90:10). The resulting mother liquors were subjected to preparative TLC on Silica gel 60 F254 using hexane:ethyl acetate (1:1) as mobile phase to obtain compound 4.

### Compound identification

The isolated compounds were identified by proton and carbon nuclear magnetic resonance (^1^HNMR) and ^13^CNMR, (Bruker Avance 500) (500 MHz, in CDCl_3_), heteronuclear single quantum correlation (HSQC), heteronuclear multiple bond correlation (HMBC), correlated spectroscopy (COSY), electron impact mass spectrometry (EI-MS) (Thermo Scientific EM/DSQII), infrared spectroscopy (IRS) (Bruker FT-IR IFS 25) and by comparison with literature data and reference compounds. The purity of isolated compounds was checked by HPLC/DAD using a Varian Pro Star instrument equipped with a reversed-phase column Phenomenex–KinetexXB-C18 (250 mm x 4.6 mm and 5 μdp), a Rheodyne valve (20 μL) and a photo diode array detector set at 210 nm. The chromatographic system used was that reported by Laurella et al. [23].

### HPLC analysis of *M*. *micrantha* and *M*. *variifolia* dichloromethane extract and quantification of bioactive compounds

For HPLC analysis, samples were eluted with a gradient of water (A) and acetonitrile (B) from 100% A to 0% A in 60 min and back to initial conditions. A flow rate of 1 mL/min was employed and the separation was done at room temperature (18–25°C). Data were analysed with Varian Star 5.5 (USA). *Mikania micranta* and *M*. *variifolia* organic extracts and pure isolated compounds (used as reference) were dissolved in methanol and water (9:1) at a concentration of 5 mg/mL and 1 mg/mL respectively. Water employed to prepare working solution was of ultrapure quality (Milliq). Methanol and acetonitrile (HPLC) J.T. Baker were used.

### *In vitro* trypanocidal activity

The evaluation of *T*. *cruzi* epimastigotes growth inhibition was performed by the [^3^H] thymidine uptake assay [25]. Briefly, exponentially growing epimastigotes were adjusted to a cell density of 1.5 × 10^6^ parasites/mL in fresh medium. Parasites were allowed to grow for 72 h at 28°C in medium with or without different concentrations of each compound (0–5 μg/mL) in triplicate. Percent of inhibition was calculated as {100 − [(cpm of treated parasites/ cpm of untreated parasites) × 100]}. The compound concentration at which the parasite growth was inhibited by 50% (IC_50_) was determined after 72 h. Benznidazole was used as positive control.

The trypanocidal effect of the purified compounds was also tested on bloodstream trypomastigotes. Briefly, mouse blood containing trypomastigotes was diluted in complete RPMI -1640 Medium (Sigma-Aldrich R6504- Batch 029K83102) with 10% (vol/vol) fetal calf serum (FCS) (Internegocios) to adjust the parasite concentration to 3 × 10^5^/mL. Parasites were seeded in duplicate in a 96-well microplate in the presence of each compound (0–50 μg/mL) or controls and incubated at 4 °C for 24 h. The number of remaining living parasites in each sample was determined in 5 μL of cell suspension diluted 1/5 in lysis buffer (0.75% NH_4_Cl, 0.2% Tris, pH 7.2) and counted in a Neubauer chamber. Benznidazole was used as positive control.

The effect of each compound on intracellular forms of *T*. *cruzi* was assayed using β-galactosidase transfected parasites [25]. Briefly, 96-well plates were seeded with Raw 264.7 cells at 5x10^3^ per well in 100 mL of culture medium and incubated for 24 h at 37 °C in a 5% CO_2_ atmosphere. Cells were washed and infected with transfected blood trypomastigotes expressing β-galactosidase at a parasite/cell ratio of 10:1. After 12 h of co- culture, plates were washed twice with PBS to remove unbound parasites and each pure compound was added at different concentrations (0.001–50 μg/mL) in 150 μL of fresh complete RPMI medium without phenol red (Gibco, Rockville, MD). Controls included infected untreated cells (100% infection control) and uninfected cells (0% infection control). The assay was developed 5 days later by the addition of chlorophenol red-β-D-galactopyranoside (CPRG) (100 mM) and 1% Nonidet P40. Plates were then incubated for 4–6 h at 37 °C and the absorbance was measured at 595 nm in a microplate reader (Bio-Rad Laboratories, Hercules, CA). The percentage inhibition was calculated as 100–{[(absorbance of treated infected cells)/(absorbance of untreated infected cells) x 100} and the IC_50_ value was estimated. Benznidazole was used as positive control.

### *In vitro* leishmanicidal activity

The growth inhibition of *Leishmania braziliensis* promastigotes was evaluated by the MTT method. Parasites (5x10^6^) were settled at a final volume of 150 μL in a flat-bottom 96-well microplate and cultured at 37°C in a 5% CO_2_ atmosphere in the absence or presence of increasing concentrations of the pure compounds. After 72 h, 3-(4,5- dimethylthiazol-2-yl)-2,5-diphenyltetrazolium bromide (MTT) was added at a final concentration of 1.5 mg/mL. Plates were incubated for 2 h at 37°C. The purple formazan crystals formed were completely dissolved by adding 150 μL of ethanol and the absorbance was read at 595 nm in a microplate reader. The inhibition percentage was calculated as {100 − [(DO_595nm_ of treated parasites/ DO_595nm_ of untreated parasites) × 100]}. Compounds were tested at 0–50 μg/mL. Amphotericin B was used as positive control.

### Host cell toxicity

The human monocyte leukemia THP1 (ATCC TIB202) cell line were obtained from the vendor. Cells were thawed, expanded and settled at a concentration 5x10^5^ in a final volume of 150 μL in a flat-bottom 96-well microplate and cultured at 37°C in a 5% CO_2_ atmosphere in the absence or presence of increasing concentrations of the pure compounds. After 24 h, the cell viability was determined by the trypan blue exclusion method in the absence and presence of increasing concentrations of the compounds [26]. The 50% cytotoxic concentration (CC_50_) and the selectivity index (SI = CC_50_/IC_50_) were determined for each compound for *T*. *cruzi* trypomastigotes and amastigotes.

### Cytokine production

RAW 264.7 (ATCC TIB71) macrophage cell line were obtained from ATCC and cultured (1×10^5^) in duplicate in 24-well plates in the presence of 2.5 or 25 μg/mL of Compound 3, LPS (10 μg/mL) plus INF-γ (0.30 μg/mL) (positive control), or medium alone (negative control). After 24 h, supernatants were collected and stored at -80 °C until cytokine analysis. IL-12 and TNF-α levels were determined by sandwich ELISA (BD Pharmingen, and R&D System, Minneapolis, MN, respectively) according to manufacturer’s instructions. Supplied standards were used to generate a standard curve.

### Animal model of *T*. *cruzi* infection

Inbred male Balb/c mice were nursed at the Departamento de Microbiología, Facultad de Medicina, Universidad de Buenos Aires. Groups of six Balb/c male mice (6 to 8 weeks old) were infected with 1000 bloodstream *T*. *cruzi* trypomastigotes by the intraperitoneal route. Five days after infection, the presence of circulating parasites was confirmed by the microhematocrit method (Sülsen et al., 2013). Mice were treated daily with compound 3 or benznidazole (1 mg/kg of body weight/day) for five consecutive days (days 4 to 8 post-infection) by the intraperitoneal route. Drugs were resuspended in DMSO and then adjust to concentration in 0.1 M phosphate buffered saline (PBS, pH 7.2); this vehicle was also employed as a negative control. Levels of parasitemia were monitored every 2 days in 5 μL of blood diluted 1:5 in lysis buffer (0.75% NH_4_Cl, 0.2% Tris, pH 7.2) by counting parasites in a Neubauer chamber. The number of animal deaths was recorded daily.

The weight of each animal was evaluated during the acute phase of infection. Results were expressed as the ratio between the weight on each day and the weight registered the day of the infection multiplied by 100.

### Ethics statement

Animal experiments were approved by the Review Board of Ethics of Universidad de Buenos Aires, Facultad de Medicina (Argentina), with the No 2943/2013 and conducted in accordance with the Guide for the Care and Use of Laboratory Animals of the National Research Council [27].

### Statistical analysis

Results are presented as means ± SEM. GraphPad Prism 5.0 software (GraphPad Software Inc., San Diego, CA) was employed to carry out calculations. To calculate the IC_50_ values, the percentages of inhibition were plotted against the drug concentration and fitted with a straight line determined by a linear regression (Sigma Plot 10 software). Results presented are representative of three to four independent experiments.

Parasitemia and weight loss were analyzed using a non parametric test: Mann-Whitney test. The survival curves were analyzed with a log rank test.

The statistical significance was determined by one-way analysis of variance (ANOVA) performed with the GraphPad Prism 5.0 software (GraphPad Software Inc., San Diego, CA). Comparisons were referred to the control group. P values <0.05 were considered significant.

## Results

### Trypanocidal activity and fractionation of *Mikania variifolia* dichloromethane extract

The Mikania *variifolia* dichloromethane extract was evaluated for its trypanocidal activity on *T*. *cruzi* epimastigotes. At 100 μg/mL, the extract caused a 97.2% of inhibition of parasite growth. Two active fractions were obtained by bioassay-guided fractionation which caused inhibitions of 99.3% and 96.1%, respectively at 10 μg/mL. Three compounds (1–3) were isolated from these fractions.

### Isolation and identification of compounds from fractions 3 and 4 of *Mikania micrantha*

**Fig 1:**
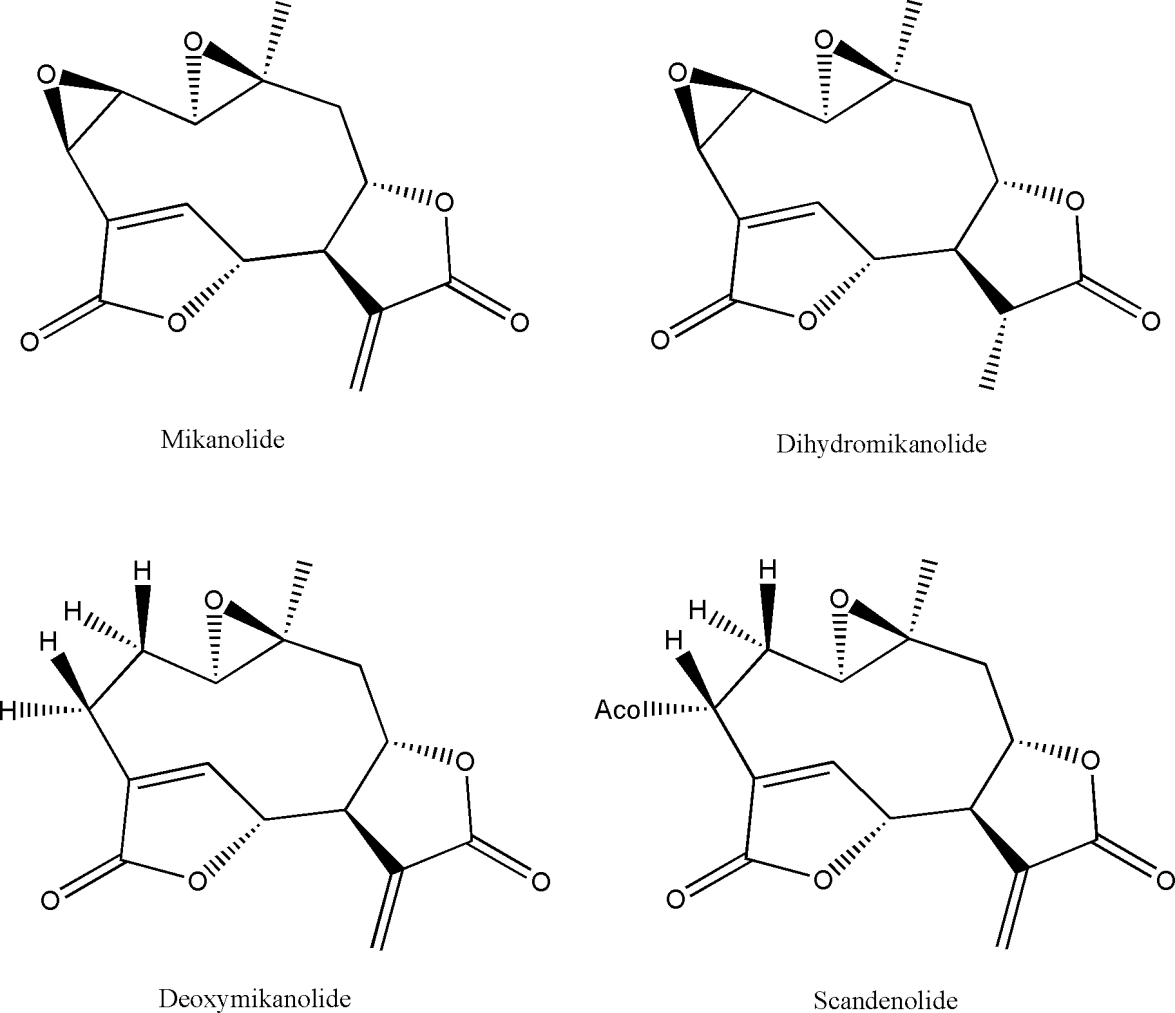
Chemical structures of mikanolide, dihydromikanolide, deoxymikanolide and scandenolide.

From the active fractions (MM3 and MM4) of the organic extract of *Mikania micrantha*, four compounds (1–4) were isolated. Compounds 1–4 were identified by spectroscopic methods and by comparison with literature data [18]. Compound 1 was identified as mikanolide; compound 2 as dihydromikanolide, compound 3 as deoxymikanolide and compound 4 as scandenolide (Fig 1).

### Quantification of mikanolide and deoxymikanolide in *M*. *micrantha* and *M*. *variifolia* dichloromethane extracts

Two major peaks (rt = 21.4 and 19.9 min) appeared in the HPLC chromatogram of *M*. *micrantha* and *M*. *variifolia* dichloromethane extracts, corresponding to mikanolide and deoxymikanolide, respectively. The content of these sesquiterpene lactones is shown in Table 1.

**Table 1:** Quantification of deoxymikanolide and mikanolide in *M*. *micrantha* and *M*. *variifolia* extracts.

Compounds	Retention time (min)	Content (%) *	
		*M*. *micrantha*	*M*. *variifolia*
Deoxymikanolide	19.9	6.4	0.4
Mikanolide	21.4	21.0	2.2

* calculated on dichloromethane extract dry weight

### *Trypanosoma cruzi* growth inhibition by isolated compounds

**Fig 2:**
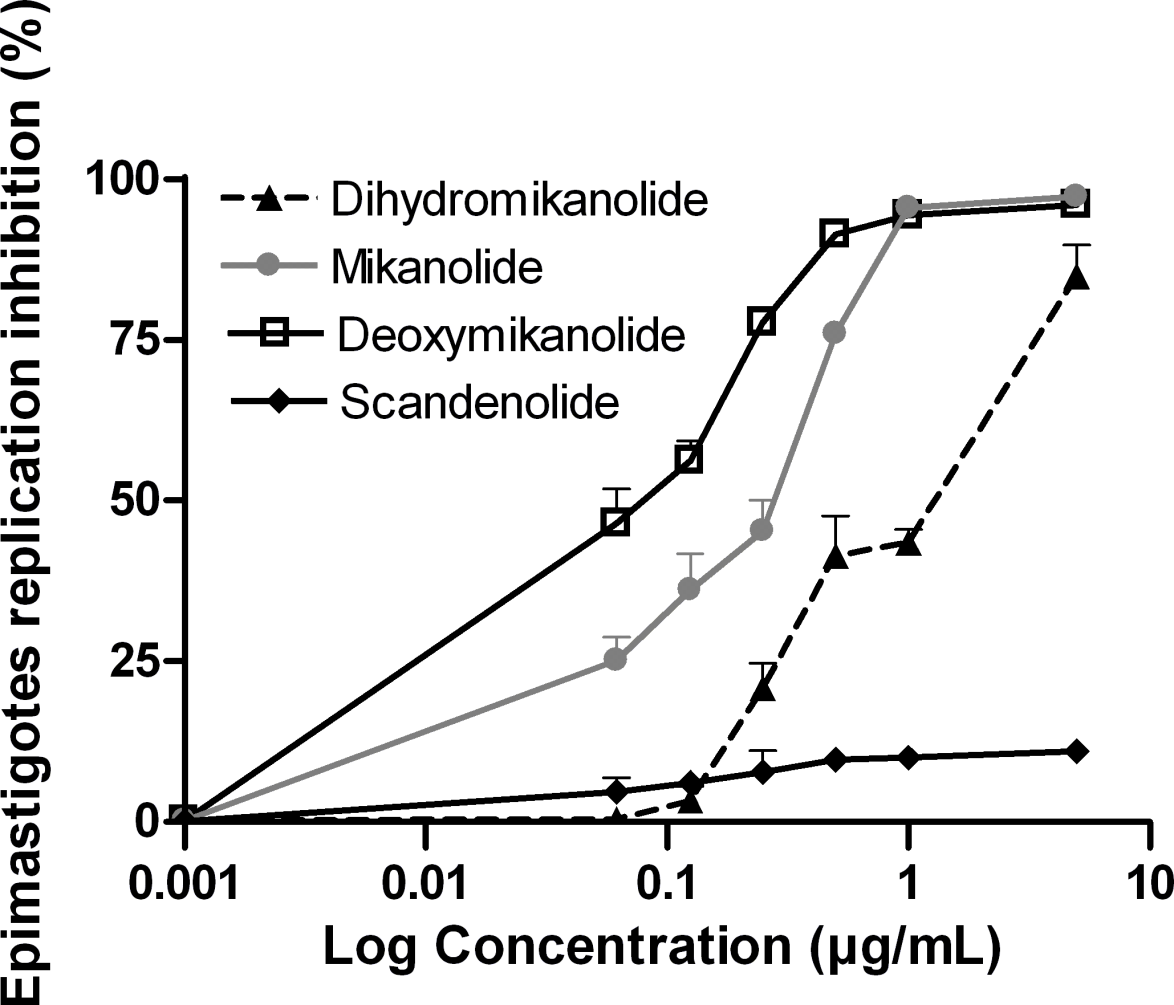
Inhibition of *T*. *cruzi* epimastigotes by mikanolide, dihydromikanolide, deoxymikanolide and scandenolide. Epimastigotes were adjusted at 1.5x10^6^ parasites/mL and cultured for 72 h at 28°C in the presence of the compounds at a final concentration ranging from 0–50 μg/mL. Growth inhibition of parasites was evaluated by a [^3^H] thymidine uptake assay. The percentage of inhibition was calculated as 100-[(cpm of treated parasites)/(cpm of untreated parasites)]x100. Values represent mean ± SEM from three independent experiments carried out in triplicate.

The trypanocidal activity of the isolated compounds was analyzed on *T*. *cruzi* epimastigotes. All the compounds were active, with 50% inhibitory concentration (IC_50_) values of 0.08, 0.7 and 2.5 μg/mL, for deoxymikanolide, mikanolide and dihydromikanolide, respectively. Conversely, scandenolide was not active against epimastigotes (IC_50_ = 137.2 μg/mL) (Fig 2). For benznidazole, an IC_50_ of 1.7 μg/mL was registered (S1 Fig).

**Fig 3:**
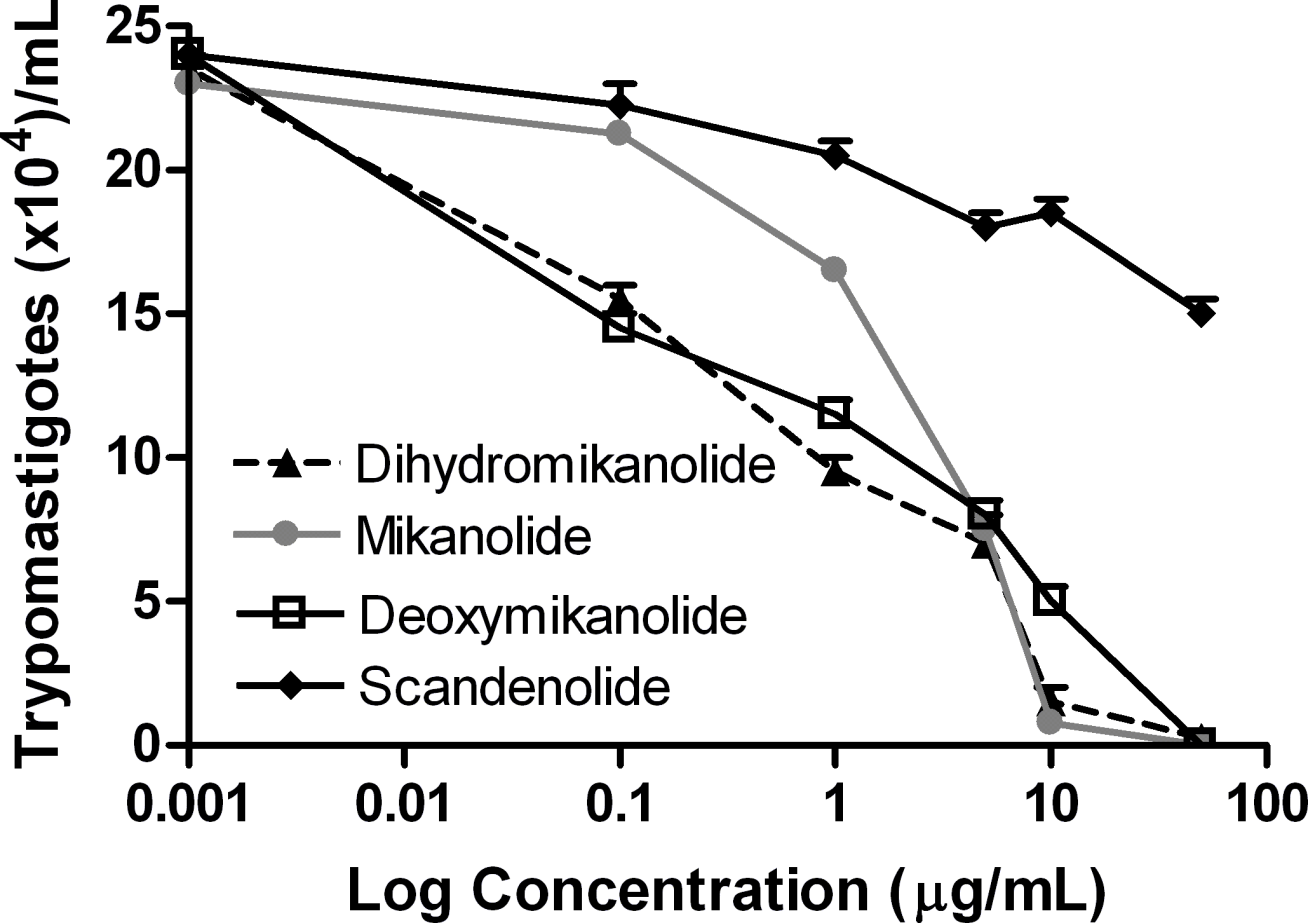
Trypanocidal activities against *T*. *cruzi* trypomastigotes of mikanolide, dihydromikanolide, deoxymikanolide and scandenolide. Bloodstream trypomastigotes were cultured in duplicate in the presence of 0 to 50 μg/mL of mikanolide, dihydromikanolide, deoxymikanolide and scandenolide. Cultures were done in 96-well plates with 3 x 10^5^ parasites/mL during 24 h, and the remaining live parasites were counted in a Neubauer chamber. Symbols represent mean ± SEM.

The trypanocidal activity against bloodstream trypomastigotes was then analyzed for each drug. As shown in Fig 3, an important decrease in the remaining live parasite count was observed for dihydromikanolide, deoxymikanolide and mikanolide, in comparison with controls (IC_50_ = 0.3, 1.5 and 2.1 μg/mL, respectively). The reference drug (benznidazole) showed an IC_50_ of 10.4 μg/mL (S1 Fig).

**Fig 4:**
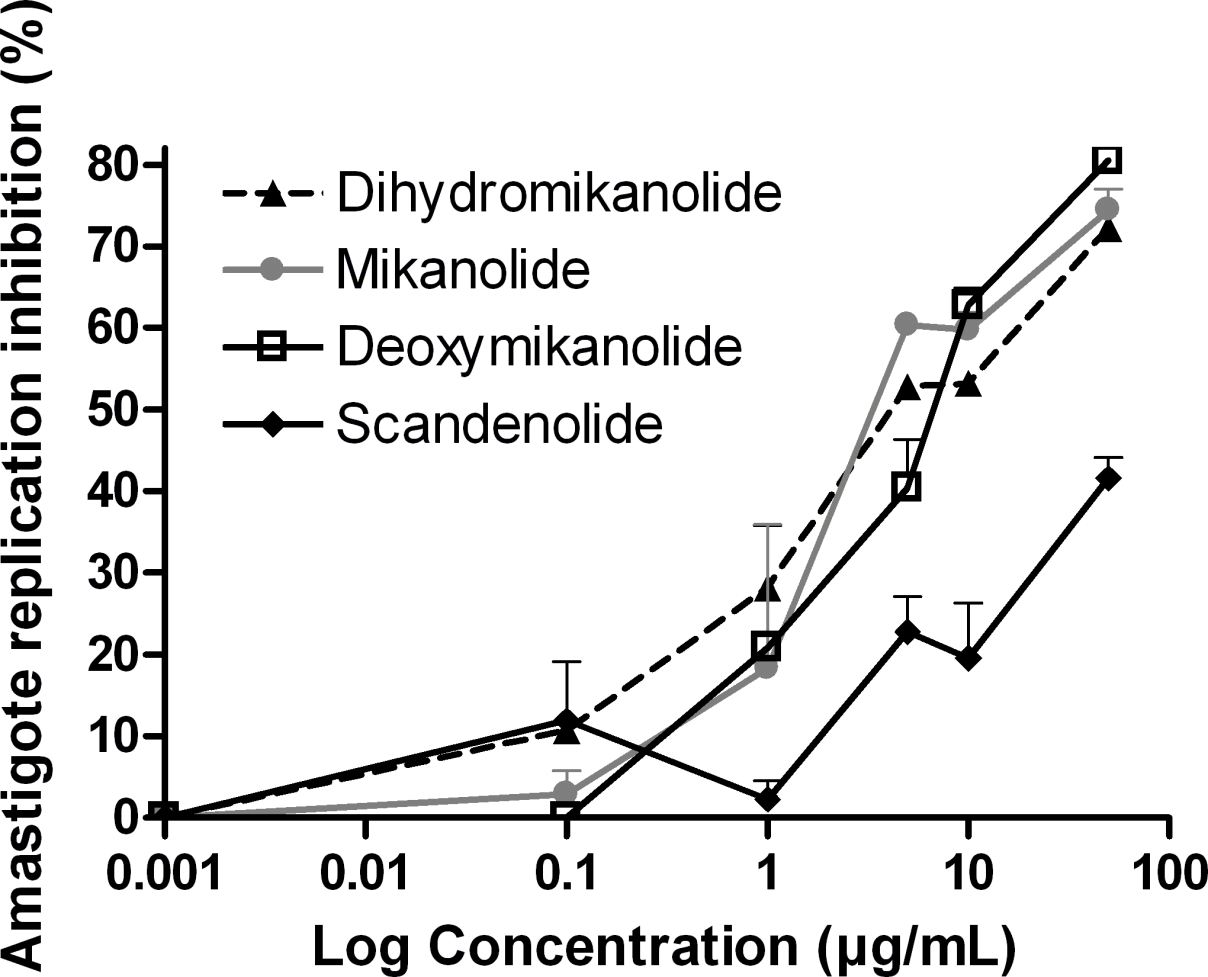
Inhibition of *T*. *cruzi* amastigotes by mikanolide, dihydromikanolide, deoxymikanolide and scandenolide. Mammalian cells (5x10^3^ cells/well) were seeded in 96 well plates and infected 24 h later with transfected trypomastigotes expressing β-galactosidase. After 24 h of co-culture, plates were washed and compounds were added at 0–50 μg/mL in 150 μL medium. On day 6 post-infection, the assays were developed by the addition of CPRG (100 mM) and Nonidet P-40 (1%). Plates were incubated for 6 h and quantified at 570 nm. Controls included infected untreated cells (100% infection control). The percentage of inhibition was calculated as 100-{[(Absorbance of treated infected cells)/(Absorbance of untreated infected cells)]x100}.

Finally, the inhibition of amastigotes replication was tested on transgenic *T*. *cruzi* expressing the β-galactosidase. Mikanolide, deoxymikanolide and dihydromikanolide were active against this replicative form of the parasite with IC_50_ values of 4.5, 6.3 and 8.5 μg/mL, respectively (Fig 4). The IC_50_ value for benznidazole was 1.1μg/mL.

Scandenolide showed IC_50_ values of 68.4 and 60.6 μg/mL on trypomastigotes and amastigotes, respectively, therefore it was not active against this replicative form.

### Antileishmanial activity of isolated compounds

**Fig 5:**
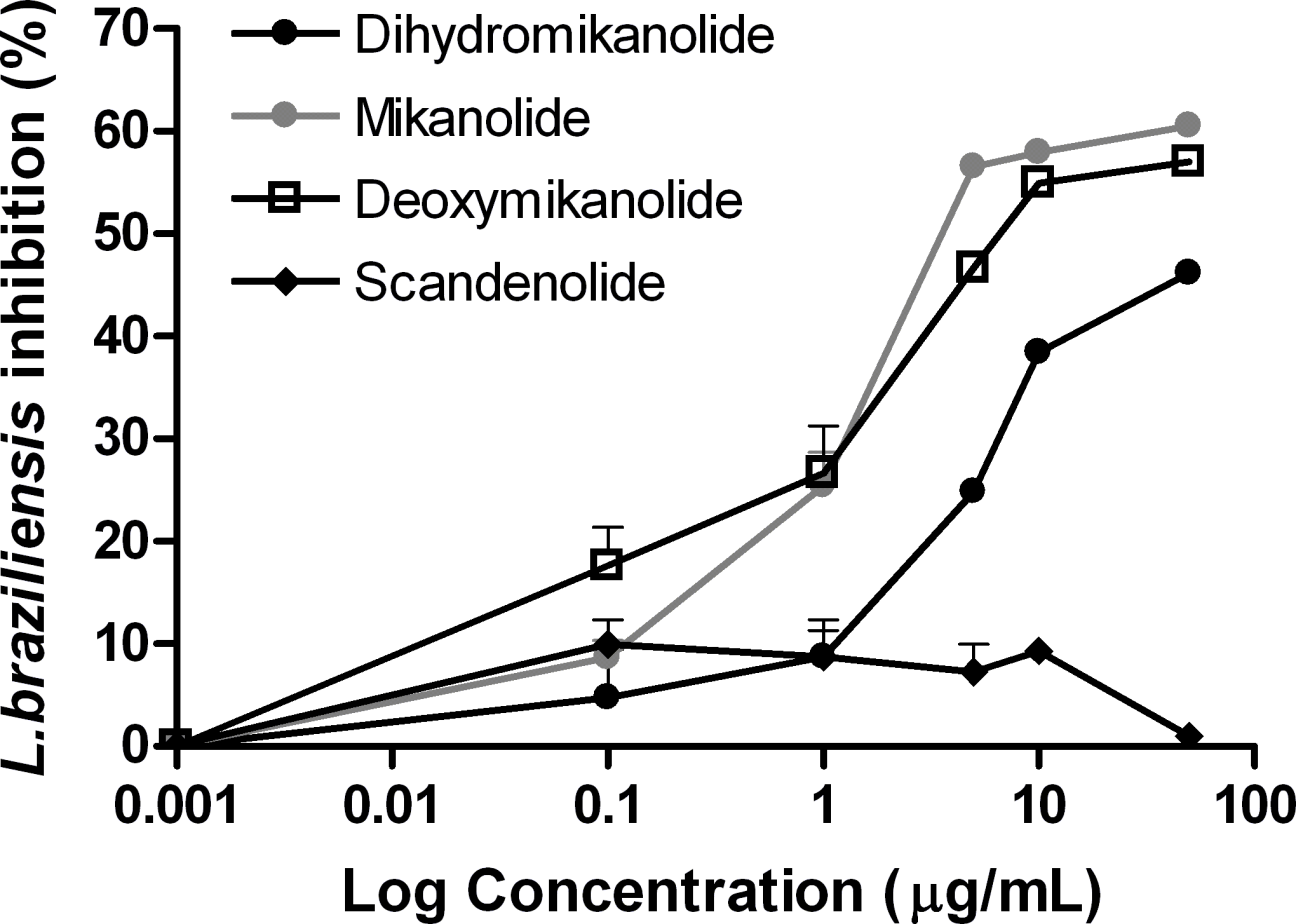
Leishmanicidal activity on *L*. *braziliensis* promastigotes of mikanolide, dihidromikanolide, deoxymikanolide and scandenolide. Parasites were cultured for 72 h in the presence of the compounds (0–50 μg/mL). Growth inhibition of parasites was evaluated by a MTT assay. The percent of inhibition was calculated as {100 − [(DO_595nm_ of treated parasites/ DO_595nm_ of untreated parasites) × 100]}. Values represent mean ± SEM from three independent experiments carried out in triplicate.

A moderate activity against *L*. *braziliensis* promastigotes was observed with mikanolide, deoxymikanolide and dihydromikanolide (IC_50_ values of 5.1, 11.5 and 57.1 μg/mL, respectively). By contrast, no activity was registered for scandenolide (IC_50_ = 252.0 μg/mL) (Fig 5). The reference drug, amphotericin B, presented an IC_50_ value of 0.26 μg/mL (S2 Fig).

### Cytotoxicity

The *in vitro* cytotoxic effect of each compound was evaluated on a human cell line by the trypan blue exclusion method. Viable human cells were incubated in the absence and presence of increasing concentrations of the compounds. Deoxymikanolide presented a CC_50_ value of 79.37 μg/mL, being the SI for *T*. *cruzi* trypomastigotes and amastigotes 54 and 12.5, respectively. These SI values were higher than those obtained for mammalian cells. CC_50_ values for dihydromikanolide and mikanolide were 12.98 and 22.3 μg/mL, respectively. Selectivity indexes for these STLs were 50 and 1.52 (dihydromikanolide), and 10.66 and 4.96 (mikanolide), for trypomastigotes and amastigotes, respectively. Selectivity indexes of the compounds for *L*. *braziliensis* were lower than 10 (Table 2).

**Table 2:** Cytotoxicity on human monocyte leukemia THP1 and selectivity indexes of the isolated compounds.

Compounds	CC_50_ (μg/mL)	SI		
		*T*. *cruzi* trypomastigotes	*T*. *cruzi* amastigotes	*L*. *braziliensis* promastigotes
**Mikanolide**	22.3	10.7	4.3	4.4
**Deoxymikanolide**	79.4	54.0	12.5	6.9
**Dihydromikanolide**	13.0	49.9	1,5	0.2
**Scandenolide**	863.6	12.6	14.2	3.4

### Treatment of *Trypanosoma cruzi* infected mice with deoxymikanolide

**Fig 6:**
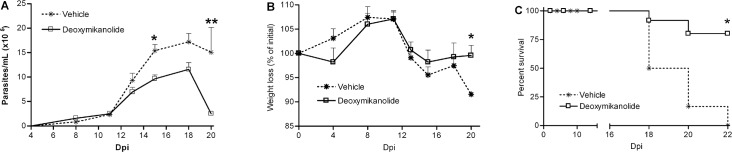
*In vivo* deoxymikanolide trypanocidal activity. Balb/c mice infected with a lethal dose of *T*. *cruzi* were treated for 5 consecutive days (days 4 to 8 post-infection) with either deoxymikanolide or with vehicle (phosphate buffered saline). Parasitemia (A), body weight loss (B), and Survival (C), during the acute phase of the parasite infection were analyzed. Results presented are representative of three independent experiments. *p = 0.028, Mann- Whitney test.

Since deoxymikanolide presented good selectivity on differents *T*. *cruzi* stages, this compound was selected for an *in vivo* study. Balb/c mice were infected with a lethal dose of *T*. *cruzi* (RA strain) and treated for 5 consecutive days with either deoxymikanolide or the vehicle. As shown in Fig 6A, infected mice that received deoxymikanolide presented a lower blood parasitemia, as compared to controls. Moreover, in terms of area under the parasitemia curve, a 1.5-fold reduction was observed in deoxymikanolide-treated mice with respect to controls; whereas a 2.3-fold was observed for benznidazole-treated mice (S1 Fig). Interestingly, the treatment with deoxymikanolide was able to reduce the weight loss observed during the acute phase of infection, as compared to control mice (*p* = 0.028, Mann-Whitney test) at 20 days post infection (dpi) (Fig 6B). More importantly, in deoxymikanolide-treated mice, a significant decrease in the mortality caused by *T*.*cruzi* infection was observed (*p* = 0.02, Log-rank test). While nearly 70% of deoxymikanolide-treated mice survived the acute phase of infection, 100% mortality was observed in control mice by day 22 post-infection (Fig 6C). These results highlight the *in vivo* efficacy of deoxymikanolide to induce the killing of circulating trypomastigotes during the acute infection.

### Cytokine production

**Fig 7:**
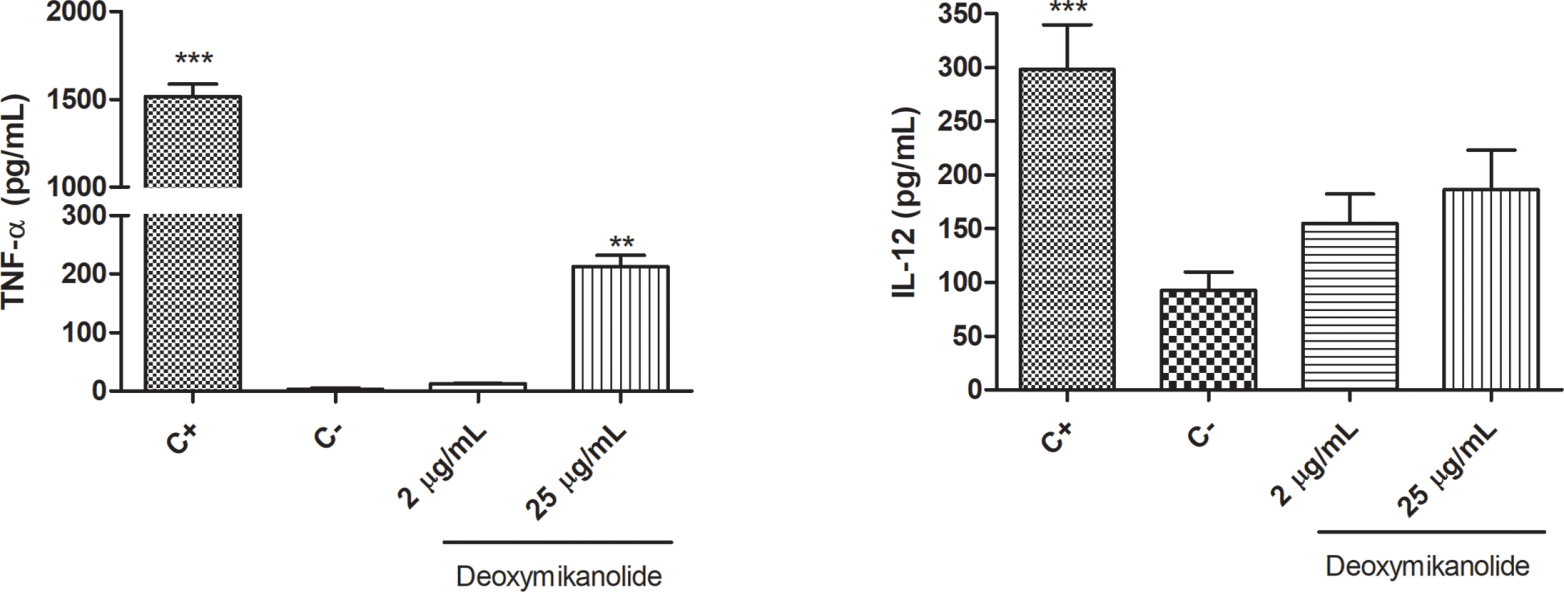
Cytokine secretion induced by deoxymikanolide on macrophages. Cells (1×10^5^cells/mL) were cultured in the presence of deoxymikanolide (2 and 25 μg/mL) for 24 h. Cells were incubated with LPS (1 μg/mL) plus INF-γ (1000 pg/mL) as positive control of macrophage IL-12 and TNF-α production. Cytokines were assayed by ELISA. Data are expressed as mean ± SEM and are representative of two independent experiments.

We further studied *in vitro* the ability of deoxymikanolide to modulate the host’s immune response, particularly, macrophage cells, which are the first line of defense against *T*. *cruzi* infection. Upon macrophage stimulation with 25 μg/mL deoxymikanolide, a significant increase in the secretion of TNF-α was observed as compared with the negative control (*p<0*.*01*). Moreover, a slight increase in the IL-12 production was observed when deoxymikanolide was used at 2 μg/mL and a higher production rate was observed when this STL was tested at 25 μg/mL (Fig 7).

## Discussion

Four sesquiterpene lactones of the germacranolide type have been isolated and identified from *M*. *variifolia* and *M*. *micrantha* organic extracts. Mikanolide, dihydromikanolide and deoxymikanolide were identified in *M*. *variifolia*, while the same compounds together with scandenolide were found to be present in *M. micrantha*. All the compounds, with the exception of scandenolide, showed high activity on *T*. *cruzi* epimastigotes. Mikanolide, deoxymikanolide and dihydromikanolide were also active against trypomastigote and amastigote forms.

Due to the existence of coinfections with both *T*. *cruzi* and *Leishmania* spp., the isolated compounds were also tested *in vitro* against *L*. *braziliensis*, the main agent of tegumentary leishmaniasis in Argentina [28]. Mikanolide and deoxymikanolide showed significant activity against *L*. *braziliensis* promastigotes, while dihydromikanolide displayed moderate activity. On the other hand, scandenolide was not active against this parasite stage.

The major determinant for the antiprotozoal activity of STLs would be the presence of an α,β- unsaturated lactone group in the sesquiterpene lactone structure [29]. Nevertheless, some of α-santonin derivatives, lacking the exomethylene group, have shown trypanocidal activity [30]. Steric and electronic factors could also influence the anti-*T*. *cruzi* activity of STLs [31].

The four isolated compounds differ from each other in the number of epoxy groups, in the presence or absence of the exocyclic double bond and in the presence of an OAc group at C-3. In comparing the results of activity obtained for mikanolide and dihydromikanolide on the different *T*. *cruzi* stages, the absence of the exocyclic double bond in dihydromikanolide with respect to mikanolide, does not seem to affect its activity against this parasite. Despite the different number of epoxy groups present in mikanolide and deoxymikanolide structures, both compounds showed similar activity on this parasite.

On the other hand, the presence of the exocyclic double bond seems to be important for the leishmanicidal activity, since mikanolide and deoxymikanolide were active, while dihydromikanolide was moderately active.

Scandenolide did not show any antiprotozoal activity, suggesting that the presence of an OAc at C-3 could be responsible for the decrease of such activity.

A strong correlation between biological activity and cytotoxicity of STLs has been reported [29]. Although many of them display a considerable cytotoxic activity against mammalian cells, some of these compounds show more selectivity against the parasites [31]. Deoxymikanolide presented a CC_50_ that was approximately four times higher than mikanolide, thus proving to be more selective for the parasite.

There are previous reports describing the presence of mikanolide, dihydromikanolide, deoxymikanolide and scandenolide in *M*. *micrantha* [18]. Although these sesquiterpene lactones have been evaluated for other activities [20, 22, 32], this is the first study that describes their trypanocidal and leishmanicidal effects. Herein, the STLs of *M*. *variifolia* have been isolated for the first time.

An optimal response to treatment of trypanosomatid diseases is strongly linked to an efficient immune response. This response is mediated by the activation of macrophages with consequent production of nitrogen and oxygen intermediates that are toxic to the parasite, as well as the activation of T helper and cytotoxic cells producing INF-γ [10, 33–36]. The biological activities of lactones, including sesquiterpenoids are largely due to their inhibitory effects on the activity of a plethora of enzymes of prokaryotic organisms and eukaryotic cells [37]. Sesquiterpene lactones have proved to be potent inhibitors of different transcription factors [38, 39]. The best recognized interference of this type of compounds is the inhibition of multiple factors within the nuclear factor-κB (NF-κB) signaling system [40, 41]. These effects are presumed to be related to molecular mechanisms determining the immune activity of sesquiterpene lactones. In that sense, it has been previously reported that a guaianolide type sesquiterpene lactone with a lactone-diol moiety stimulates production of interleukin-8 (IL-8) [42] and up-regulates the lipopolysaccharide (LPS)-induced production of pro-inflammatory cytokines such as IL-6 and tumor necrosis factor-α (TNF-α). Harmatha et al. [43], have demonstrated an increase in NO production and in cytokines such as IFN-γ, IL-1β, IL-6, VEGF and GM-CSF in a range of low concentrations of 10 nM-10 mM by a guaianolide type sesquiterpene lactone. Based on its activity and selectivity, we selected deoxymikanolide for the evaluation of its immunomodulatory activity on macrophage cells, which are the first line of defense against *T*. *cruzi* and *Leishmania* infection. The results obtained suggest that, besides the antiprotozoal activity that deoxymikanolide has *per se*, this compound could stimulate the host´s immune system through the secretion of cytokines, thus exerting a protective effect against parasite infection.

## Conclusion

Four sesquiterpene lactones, mikanolide, deoxymikanolide, dihydromikanolide and scandenolide were isolated from *M*. *variifolia* and *M*. *micrantha*. With the exception of scandenolide, all the sesquiterpene lactones showed trypanocidal and leishmanicidal activities. Deoxymikanolide was the most promising compound based on its activity, selectivity and its capacity to stimulate cytokine production. Besides, this compound was also active in a murine model of *T*. *cruzi* infection. This finding makes it an interesting lead molecule which may be useful for the development of new drugs, alone or in combination with actual therapy, for the treatment of trypanosomatid diseases, in order to reduce side effects.

## Supporting information

S1 Fig **Trypanocidal activity of the reference drug benznidazol determined by *in vitro* assays against: epimastigotes (A), trypomastigotes (B) and amastigotes (C) of *T*. *cruzi*. *In vivo* treatment with benznidazol in a murine model of *T*. *cruzi* infection (D).** Area under curve (AUC) was determined. *p<0.01, ***p<0.001.
(TIF)

S2 Fig Leishmanicidal *in vitro* activity of the reference drug amphotericin B against promastigotes of *L*. *braziliensis*. (TIF)
