# Supplementary figures and images for: Assessment of sesquiterpene lactones isolated from Mikania plants species for their potential efficacy against Trypanosoma cruzi and Leishmania sp
Source: PLoS Negl Trop Dis. 2017 Sep 25;11(9):e0005929. doi: 10.1371/journal.pntd.0005929 (PMC5629014; doi:10.1371/journal.pntd.0005929)

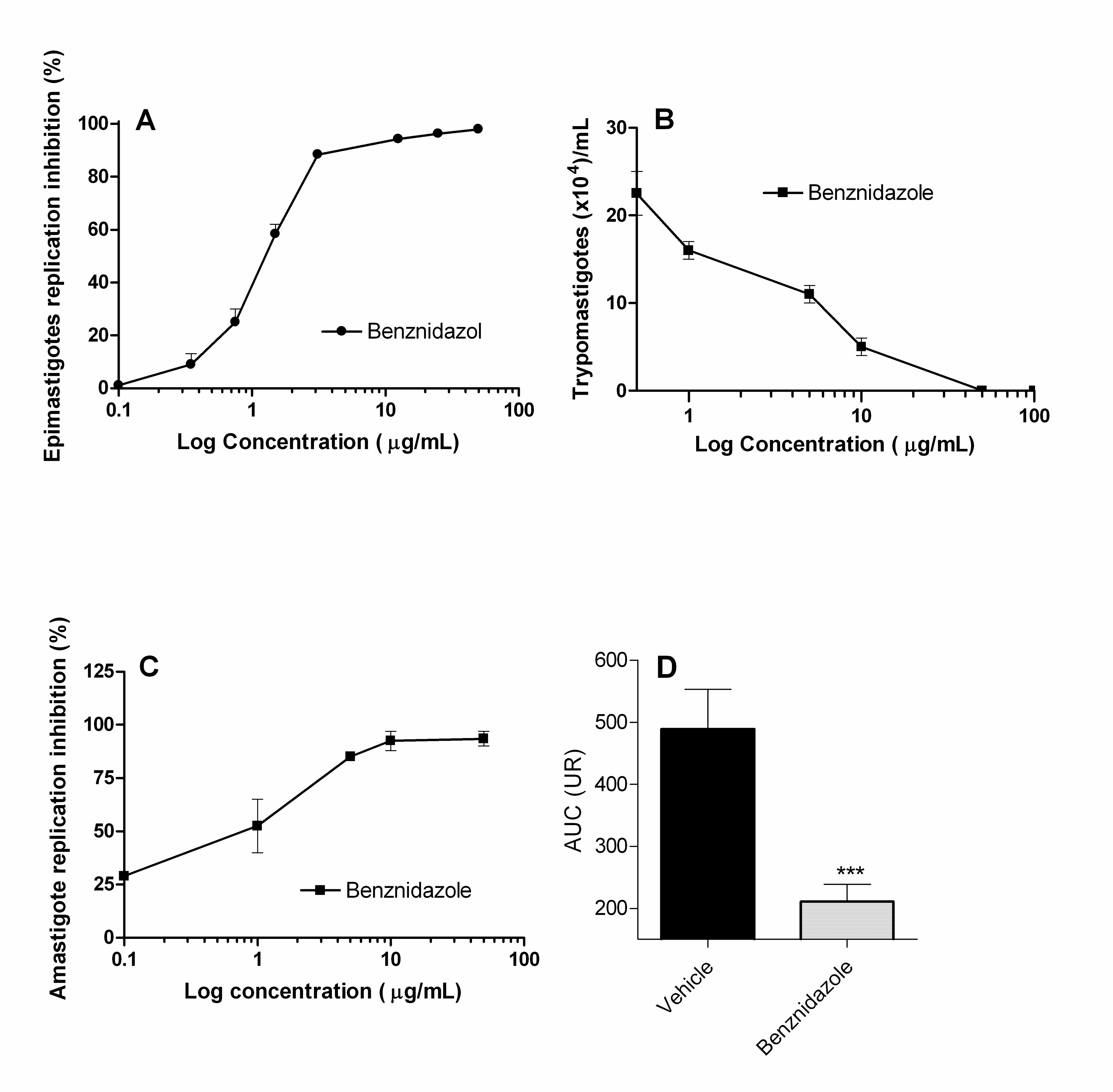

Supplement: S1 Fig — Trypanocidal activity of the reference drug benznidazol determined by in vitro assays against: epimastigotes (A), trypomastigotes (B) and amastigotes (C) of T. cruzi. In vivo treatment with benznidazol in a murine model of T. cruzi infection (D). Area under curve (AUC) was determined. *p<0.01, ***p<0.001. (TIF) [file pntd.0005929.s001.tif]

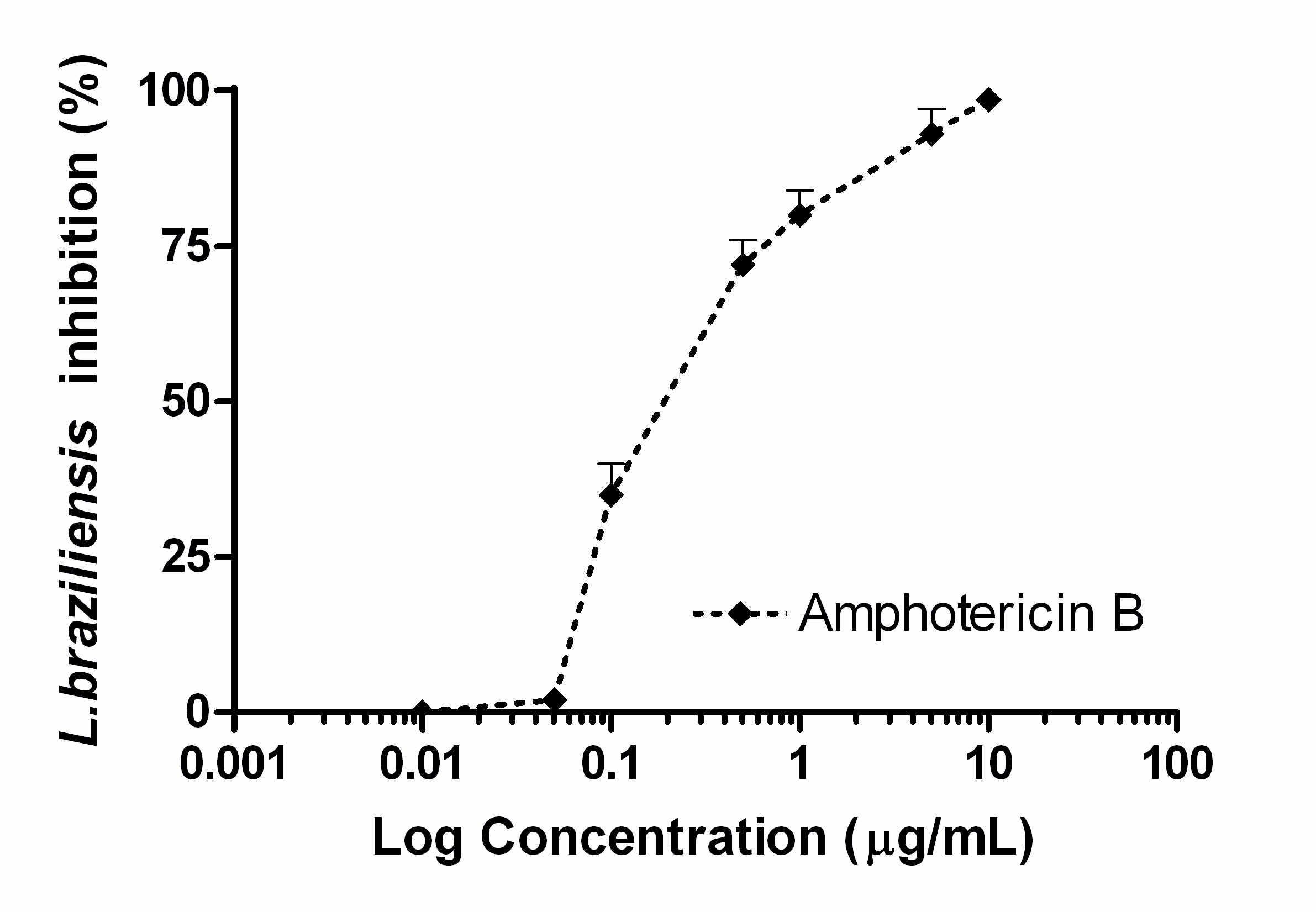

Supplement: S2 Fig — (TIF) [file pntd.0005929.s002.tif]
